# Public Health Agency of Sweden's Brief Report: Pregnant and postpartum women with severe acute respiratory syndrome coronavirus 2 infection in intensive care in Sweden

**DOI:** 10.1111/aogs.13901

**Published:** 2020-06-13

**Authors:** Julius Collin, Emma Byström, AnnaSara Carnahan, Malin Ahrne

**Affiliations:** ^1^ Public Health Agency of Sweden Solna Sweden

**Keywords:** coronavirus, coronavirus disease 2019, intensive care unit, intensive care, postpartum, pregnancy, severe acute respiratory syndrome coronavirus 2

## Abstract

The Public Health Agency of Sweden has analyzed how many pregnant and postpartum women with severe acute respiratory syndrome coronavirus 2 (SARS‐CoV‐2) infection have been treated in intensive care units (ICU) in Sweden between 19 March and 20 April 2020 compared with non‐pregnant women of similar age. Cases were identified in a special reporting module within the Swedish Intensive Care Registry (SIR). Fifty‐three women aged 20‐45 years with SARS‐CoV‐2 were reported in SIR, and 13 of these women were either pregnant or postpartum (<1 week). The results indicate that the risk of being admitted to ICU may be higher in pregnant and postpartum women with laboratory‐confirmed SARS‐CoV‐2 in Sweden, compared with non‐pregnant women of similar age.

**Abbreviations**
COVID‐19: coronavirus disease 2019
ICU: intensive care unit
PHAS: Public Health Agency of Sweden
SARS‐COV‐2: severe acute respiratory syndrome coronavirus 2
SIR: Swedish Intensive Care Registry

**Key message**

The risk of requiring intensive care may be higher in pregnant women infected with SARS‐COV‐2, or women who have recently given birth, compared with non‐pregnant women of similar age.

## 1 INTRODUCTION

In the beginning of April 2020, the Public Health Agency of Sweden (PHAS) noted that a relatively high number of pregnant and postpartum women with severe acute respiratory syndrome coronavirus 2 (SARS‐CoV‐2) infection were or had been treated in intensive care units (ICU) in Sweden. When analyzing the published literature on pregnancy and SARS‐CoV‐2, gaps in knowledge were identified, especially whether being pregnant represents a risk for increased susceptibility to infection, severity of clinical presentation and adverse outcomes for mothers and neonates.^1^, ^2^, ^3^, ^4^, ^5^, ^6^, ^7^ Through dialogue with corresponding authorities in other European countries and the USA it became apparent that none had seen a comparatively increased number of pregnant or postpartum women with SARS‐CoV‐2 infection requiring intensive care.

As a first step, PHAS analyzed how many pregnant women with SARS‐CoV‐2 infection had been treated in ICU in Sweden, compared with non‐pregnant women of similar age. This analysis was shared and discussed with the National Board of Health and Welfare and with professional medical organizations in Sweden. This is a rapid assessment of the current situation in March and April. Further analyses and research will hopefully shed more light on pregnancy and coronavirus disease 2019 (COVID‐19).

## 2 MATERIAL AND METHODS

The Swedish Intensive Care Registry (SIR)^8^ includes all cases that have received intensive care in Sweden. Most ICUs also report additional information on patients with laboratory‐confirmed SARS‐CoV‐2 as well as influenza through a special reporting module. Pregnant and postpartum women can be identified through this reporting. During the period between 19 March and 20 April, information was collected on all women aged 20‐45 years with SARS‐CoV‐2 reported through this module. Additional information on the pregnant and postpartum women was collected, such as severity of symptoms and risk factors, but because of confidentiality and the small numbers it is not currently possible to publish detailed, personal information.

For some pregnant/postpartum women, the main reason for intensive care was not symptoms of SARS‐CoV‐2 infection, but other conditions. It was not possible to ascertain whether SARS‐CoV‐2 was the primary reason for intensive care for the non‐pregnant women. Therefore, a decision was made to include the entire age group, regardless of whether SARS‐CoV‐2 was the main reason for intensive care admission, as long as the patient had laboratory‐confirmed SARS‐CoV‐2.

Population data were obtained from the Swedish population registry.^9^ On 31 December 2018, there were 1 671 740 women aged 20‐45 years in Sweden. During 2018, the Swedish Birth Registry reported 116 079 births (at ≥27 weeks of gestation). An assumption was made that births were equally distributed throughout the year, resulting in an average of 318 deliveries per day (24 hours). The gestational age was assumed to be 40 weeks from the last menstrual period, on average. This resulted in an estimate that 84 913 women were likely to be pregnant in Sweden on any given day. Thus, between 19 March and 20 April 2020, 95 089 women were estimated to have been pregnant at some point.

Three sensitivity analyses were also performed. As the estimate described above only includes pregnancies from gestational age 27 weeks, there is a degree of under‐ascertainment regarding the number of pregnancies, because miscarriages and early stillbirths are not included. To compensate for this, a 50% higher value for the number of pregnancies was used in the first sensitivity analysis, based on a miscarriage rate of 28% (ranging from 10% at 20 years of age to 40% above 35 years of age),^10^ to be sure to be well above this rate. This may be an unrealistically high number of pregnancies, but it was adopted to avoid an overestimation of risk while interpreting the results.

In the second sensitivity analysis, the number of women requiring ICU was reduced to contain only those who received invasive mechanical ventilation, to account for the possibility of a slightly lower threshold for admitting pregnant women to ICU as a precaution. The third sensitivity analysis combined the aspects of the first and second sensitivity analyses.

For comparison and to obtain a picture of the need for intensive care during an epidemic, the numbers of pregnant women and women in the same age group reported in intensive care with laboratory‐confirmed influenza during the 2015‐2016 influenza season (week 40 of 2015 to week 20 of 2016) were analyzed. These data were analyzed together with the number of pregnant women in the population during the same period, using the same approach as in the first sensitivity analysis. The 2015‐2016 seasonal influenza epidemic was dominated by influenza A(H1N1)pdm09.^11^

### 2.1 Ethical approval

This study was completed as part of PHAS responsibility for public health issues at a national level and its subsequent work on surveillance of COVID‐19 during the pandemic and was exempt from formal ethical approval.

## 3 RESULTS

In total, 53 women aged 20‐45 years with SARS‐CoV‐2 admitted in ICU were reported during the period between 19 March and 20 April. Thirteen of these women were pregnant (n = 11) or had recently given birth (n = 2) on admission (within 1 week postpartum). Their age varied between 20 and 35 years, and gestational age varied between 13 and 40 weeks of gestation. Risk factors reported for some of the women were gestational diabetes and obesity. For seven women, outcome of the pregnancy was known and of these, five had delivered the baby by cesarean section. The indication for cesarean section was not known in detail for all, but for two, the indication reported was obstetric and for two, the indication reported was SARS‐CoV‐2 symptoms.

All of the pregnant or postpartum women required intensive care. In addition, 7 of the 13 women required invasive mechanical ventilation. All of the women have been discharged from the ICU with a median stay of 6 days in intensive care (range <1‐21 days). Of the 40 non‐pregnant women, 29 required invasive mechanical ventilation.

The incidence of requiring intensive care in Sweden in conjunction with laboratory‐confirmed SARS‐CoV‐2 during the study period was 14.4 per 100 000 (95% CI 7.3‐23.4) for pregnant/postpartum women and 2.5 per 100 000 (95% CI 1.8‐3.5) for non‐pregnant women in the same age group. The first sensitivity analysis, which included 146 634 pregnancies, resulted in an incidence of intensive care with laboratory‐confirmed SARS‐CoV‐2 of 9.1 per 100 000. When only including the cases requiring invasive mechanical ventilation, the incidences of invasive mechanical ventilation in ICU with laboratory‐confirmed SARS‐CoV‐2 among pregnant/postpartum women and non‐pregnant women were 7.4 per 100 000 and 1.8 per 100 000, respectively.

An analysis similar to the first sensitivity analysis was performed for the 2015‐2016 influenza season, included 180 903 pregnant women, and resulted in an incidence of intensive care with laboratory‐confirmed influenza of 3.9 per 100 000, compared with 1.8 per 100 000 for non‐pregnant women. The relative risks are presented in Table 1. Relative risk indicates the increased probability of receiving intensive care in conjunction with laboratory‐confirmed SARS‐CoV‐2 for pregnant or postpartum women, compared with non‐pregnant women in the same age group.

**TABLE 1:** Relative risk of requiring intensive care for pregnant women with laboratory‐confirmed severe acute respiratory syndrome coronavirus 2 (SARS‐CoV‐2) or influenza, respectively

Population	Relative risk	95% Confidence limits	
SARS‐CoV‐2	5.39	2.89	10.08
Sensitivity analysis^1^, SARS‐CoV‐2	3.48	1.86	6.52
Sensitivity analysis^2^, SARS‐CoV‐2	4.00	1.75	9.14
Sensitivity analysis^3^, SARS‐CoV‐2	2.59	1.13	5.91
2015‐2016 seasonal influenza epidemic	2.17	0.94	4.99

^1^ 50% more pregnant women.

^2^ Only cases requiring invasive mechanical ventilation.

^3^ Sensitivity analyses 1 and 2 combined.

## 4 DISCUSSION

We identified that the risk of requiring intensive care may be higher in pregnant/postpartum women with laboratory‐confirmed SARS‐CoV‐2, compared with non‐pregnant women in the same age group, even after accounting for miscarriages and early stillbirths (<27 weeks) in the denominator. This risk was higher than that calculated for the 2015‐2016 seasonal influenza epidemic. The increased risk remained when the analysis was restricted to only those women in need of mechanical ventilation.

Our analysis has obvious limitations. It is based on a small number of pregnant and postpartum women with SARS‐CoV‐2. For some of these, SARS‐CoV‐2 symptoms were not the main reason for ICU admission, even though all had laboratory‐confirmed SARS‐CoV‐2 infection. As the same detailed information on the primary reason for intensive care was not available for non‐pregnant women, the analysis was performed without excluding those women in ICU who were not primarily admitted because of SARS‐CoV‐2. The baseline information to calculate risk is assumed and we do not have exact information. Furthermore, other confounding factors, such as pre‐existing comorbidities and socio‐economic factors, could not be analyzed in detail and analysis stratified by gestational age or different trimesters of pregnancy was not possible. Some of the pregnant women, but not all, exhibited risk factors like hypertension, overweight or obesity, and gestational diabetes. In addition, we had no details on reason for admission, and it is possible that pregnant and postpartum women are sometimes admitted for precautionary purposes. Still, the increased risk remained when only women requiring mechanical ventilation were included. The actual number of women in our analysis is very small and could reflect heightened baseline incidence rate. Our findings need to be confirmed by other studies.

Although the limitations described above need to be taken into consideration, the results generated immediate recommendations from PHAS, including suggestions on possible preventive measures. Information on pregnant women receiving intensive care in Sweden with COVID‐19 will be continuously monitored and more refined analyses will be performed. Moreover, a joint research project has been initiated to elucidate the impact of COVID‐19 during pregnancy on maternal and neonatal outcomes, using data from the Swedish Pregnancy Register,^12^ the Swedish Neonatal Quality Register, and SmiNet.^13^

## 5 CONCLUSION

The risk of requiring intensive care may be higher in pregnant women with laboratory‐confirmed SARS‐CoV‐2 in Sweden compared with non‐pregnant women of similar age. Pregnant women should be cautious considering the potential severe consequences of SARS‐CoV‐2 infection and those with additional risk factors such as overweight or obesity, hypertension and gestational diabetes should take extra precautions. This study needs to be replicated in other countries and more detailed information on symptoms, treatment, and outcomes for pregnant and postpartum women managed in ICU is needed.

## CONFLICT OF INTEREST

None declared.
